# Alantolactone inhibits cervical cancer progression by downregulating BMI1

**DOI:** 10.1038/s41598-021-87781-z

**Published:** 2021-04-29

**Authors:** Xiaodong Sun, Hongxia Xu, Tianyu Dai, Lixia Xie, Qiang Zhao, Xincai Hao, Yan Sun, Xuanbin Wang, Nan Jiang, Ming Sang

**Affiliations:** 1 Hubei Clinical Research Center for Parkinson’s Disease at Xiangyang No. 1 People’s Hospital, Hubei Key Laboratory of Wudang Local Chinese Medicine Research, Hubei University of Medicine, Shiyan, 442000 People’s Republic of China; 2 Department of Joint Surgery, Shanghai East Hospital, School of Medicine, Tongji University, Shanghai, 200092 People’s Republic of China; 3 Hubei Province Hospital of Traditional Chinese Medicine, Hubei Province Academy of Traditional Chinese Medicine, Wuhan, 430061 People’s Republic of China

**Keywords:** Cancer models, Cancer therapy, Metastasis, Oncogenes

## Abstract

Cervical cancer is the second most common cancer in women. Despite advances in cervical cancer therapy, tumor recurrence and metastasis remain the leading causes of mortality. High expression of BMI1 is significantly associated with poor tumor differentiation, high clinical grade, and poor prognosis of cervical cancer, and is an independent prognostic factor in cervical carcinoma. Alantolactone (AL), a sesquiterpene lactone, exhibits potent anti-inflammatory and anticancer activities. In this paper, we investigated the mechanism of AL in reducing the proliferation, migration, and invasion of HeLa and SiHa cervical cancer cells as well as its promotion of mitochondrial damage and autophagy. BMI1 silencing decreased epithelial-mesenchymal transformation-associated proteins and increased autophagy-associated proteins in HeLa cells. These effects were reversed by overexpression of BMI1 in HeLa cells. Thus, BMI1 expression is positively correlated with invasion and negatively correlated with autophagy in HeLa cells. Importantly, AL decreased the weight, volume, and BMI1 expression in HeLa xenograft tumors. Furthermore, the structure of BMI1 and target interaction of AL were virtually screened using the molecular docking program Autodock Vina; AL decreased the expression of N-cadherin, vimentin, and P62 and increased the expression of LC3B and Beclin-1 in xenograft tumors. Finally, expression of BMI1 increased the phosphorylation of STAT3, which is important for cell proliferation, survival, migration, and invasion. Therefore, we suggest that AL plays a pivotal role in inhibiting BMI1 in the tumorigenesis of cervical cancer and is a potential therapeutic agent for cervical cancer.

## Introduction

Globally, cervical cancer is one of the most common malignant tumors in women. Cervical cancer in China accounts for 12% of the worldwide cervical cancer incidence and for 11% of cervical cancer related deaths^1^. There are different strategies for the treatment of cervical cancer based on clinical evaluation and staging, the primary treatment is surgery or radiotherapy for early stages, whereas concomitant chemo-radiotherapy is the conventional approach in locally advanced stages^2,3^, and platinum-based drugs and taxanes are the main chemotherapy regimen for cervical cancer^4^. However, new drugs need to be developed due to drug resistance and adverse reactions observed with the current treatment^2,3,5,6^.

Alantolactone (AL) is mainly extracted from the root of *Inula helenium L*., which is a well-known traditional Chinese medicinal herb officially listed in some European pharmacopoeias as elecampane^7,8^. Inula helenium has been traditionally used in Europe as medicinal plant, is a valuable source of active compounds with anti-inflammatory activity^8,9^. Elecampane exhibits antiproliferative properties, as one of the components of traditional Tibetan medicine^10^. It belongs to a sesquiterpene lactone group. AL has anthelmintic, antifungal, anti-inflammatory, antimicrobial, and anti-proliferative effects on several cancer cell lines of the prostate, ovary, colon, and in leukemia^11–13^. However, the exact anticancer mechanism remains unclear. In this study, we investigated the inhibitory and the activation effects of AL on cell proliferation, epithelial-mesenchymal transformation, and autophagy through BMI1 inhibition. This may be useful in treating patients with cervical cancer.

BMI1 gene encodes a ring finger protein that is major component of the polycomb group complex 1 (PRC1), and performs complex functions through chromatin remodeling as an essential epigenetic repressor of multiple regulatory genes involved in embryonic development and self-renewal in somatic stem cells. This protein also plays a central role in DNA damage repair. The oncogene, BMI1 is associated with numerous cancers and resistance to certain chemotherapies when it is aberrant expression^14,15^. Overexpression of BMI1 cells in humans correlates with the advanced stage of cancer, tumor metastasis, poor prognosis, and resistance to radiation and chemotherapy^15–17^. BMI1 has also been linked with a multitude of cellular processes of immortalization, such as cell cycle progression, epithelial-to-mesenchymal transition (EMT), autophagy, induction of telomerase, and so all^18–20^.

## Results

### AL inhibits cell proliferation and promotes cell apoptosis in human cervical cancer cells

**Figure 1:**
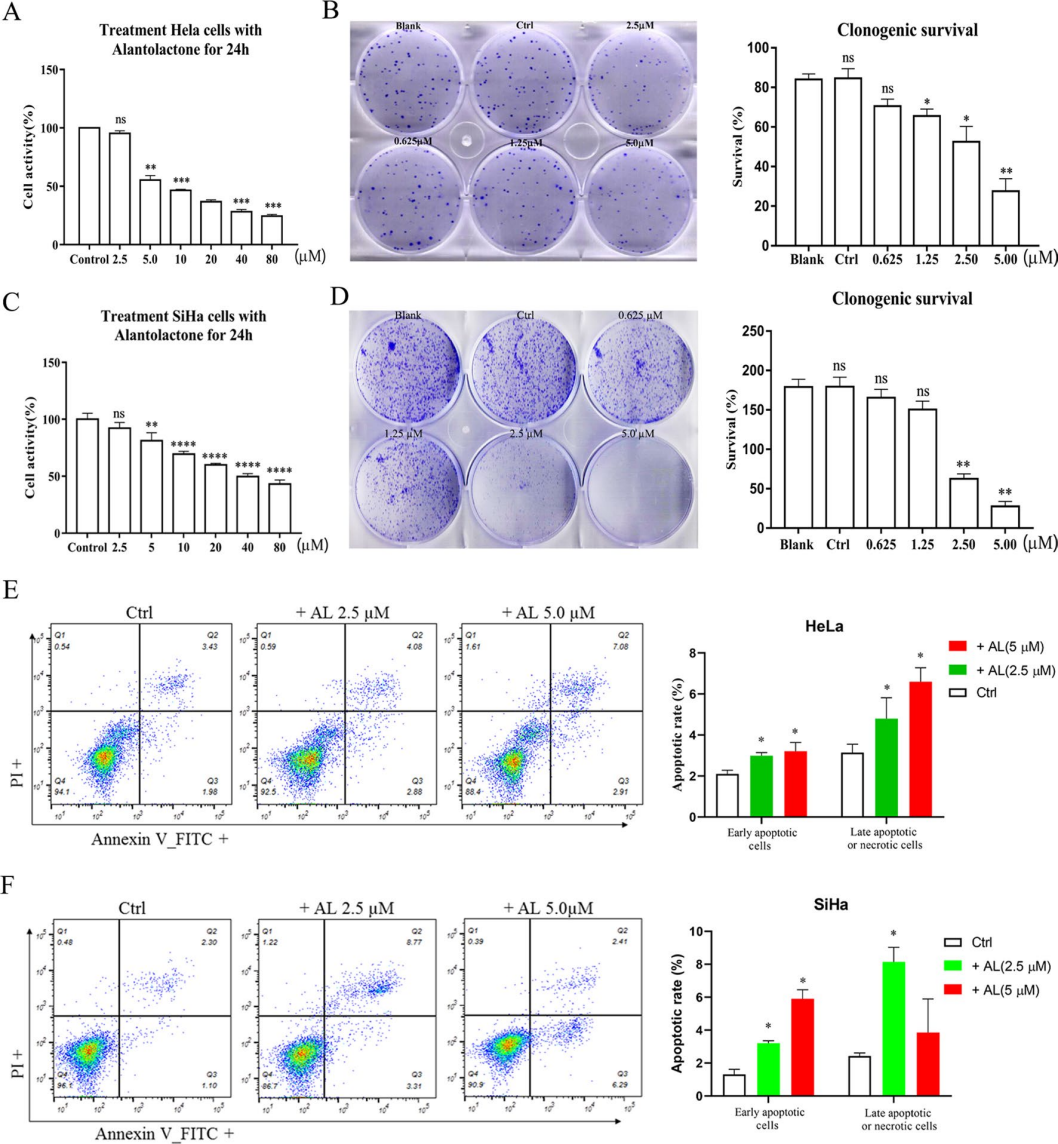
AL inhibits cell proliferation and promotes cell apoptosis in human cervical cancer cells. (**A**–**D**) AL inhibits proliferation of HeLa and SiHa cells in a dose-dependent manner. Cell proliferation was determined using CCK8 assay in HeLa (**A**) and SiHa cells (**C**), which were treated with control (vehicle), 0.625, 1.25, 2.5, 5.0, 10, 20, 40, and 80 µM AL for 24 h. Cell proliferation was determined by colony formation assay in HeLa (**B**) and SiHa cells (**D**), which were treated with blank control, control, 0.625, 1.25, 2.5, and 5.0 µM AL for 14 days. Flow cytometry using Annexin V-FITC staining shows that AL increases apoptosis of HeLa (**E**) and SiHa cells (F) in a dose-dependent manner. *P < 0.05, **P < 0.01, ***P < 0.001, ****P < 0.0001 vs control.

We assessed the anti-proliferative effects of AL in human cervical cancer cells. HeLa and SiHa cells were treated with increasing concentrations of AL (0, 2.5, 5.0, 10, 20, 40, and 80 μM) for 24 h. AL (5 μM) can significantly inhibit cell activity of HeLa and SiHa cells, and the AL IC_50_ value was 20.76 μM at 24 h in the HeLa cells, Similar effects of AL on SiHa cells were observed, and the IC_50_ value at 24 h was 37.24 μm. The cell population decreased following the concentration increase of AL (Fig. 1A,C). Moreover, an inhibitory effect was detected by colony formation assay (Fig. 1B,D). HeLa and SiHa cells were treated with different concentrations of AL for 24 h, stained with Annexin V-FITC and PI, and then measured by flow cytometry. Apoptosis rate gauged early apoptosis and late apoptosis (Fig. 1E,F). However, live cell Caspase-3 test results showed that AL has no effect on the activity of Caspase-3, which suggests that the effect of AL on cervical cancer cell apoptosis may be Caspase-independent, (Fig. S1).

### AL inhibits migration and invasion in vitro

**Figure 2:**
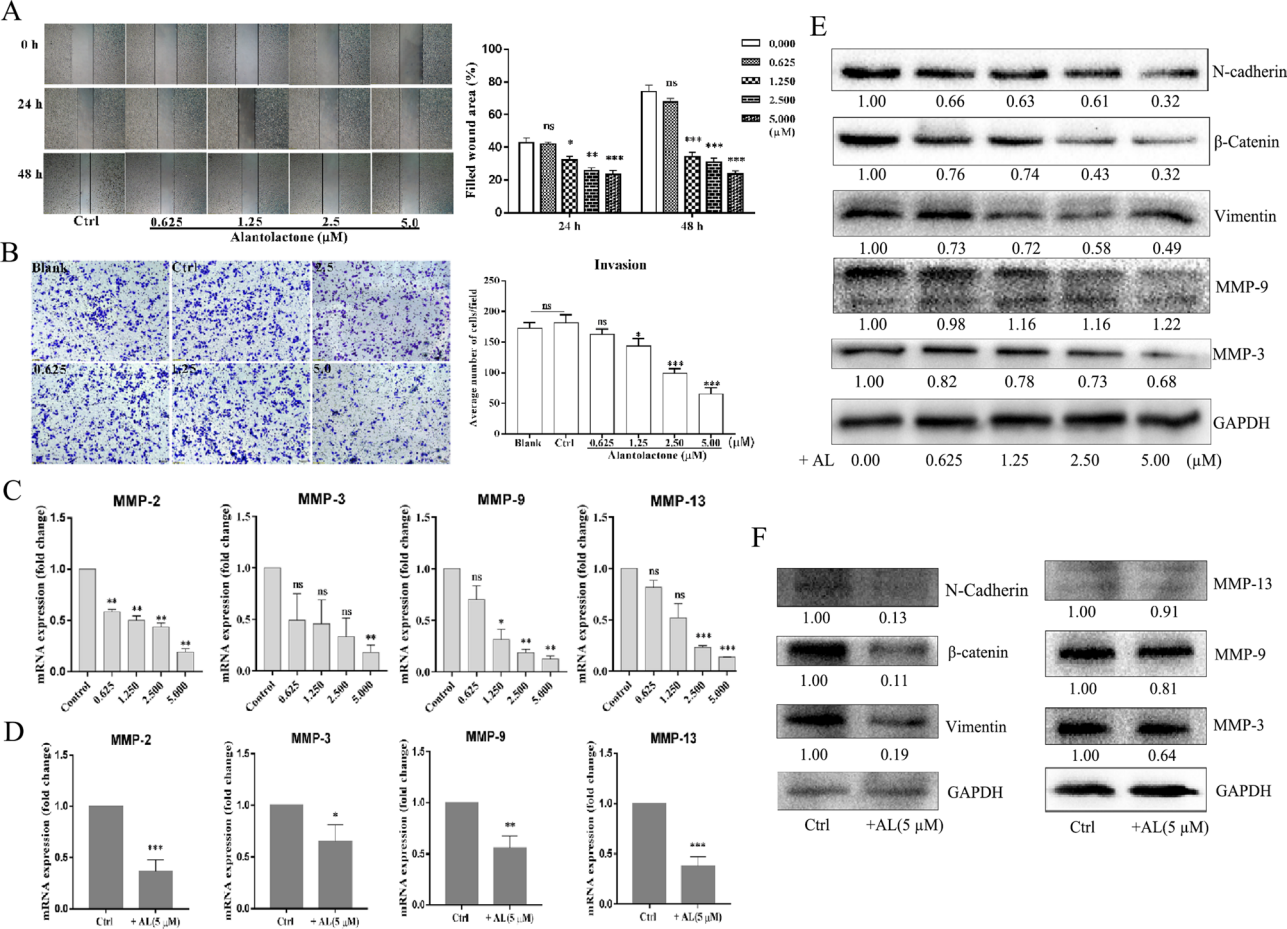
AL inhibits migration and invasion of cervical cancer cells. (**A**) The effects of AL on migration potential of HeLa cells were examined using wound healing assay. HeLa cells were seeded, scratched, and then treated with AL (0.625, 1.25, 2.5, and 5.0 μM) at indicated concentrations for 0, 24, and 48 h. The cells in the dish were examined and the scratches were measured using an optical microscope. Quantitative analysis of the scratch sizes in the wound healing assay, on the right. (**B**) The effects of AL on invasion potential of Hela cells were examined using transwell assay. Hela cells were seeded in the transwell boyden chambers and then treated with AL at indicated concentrations for 24 h. The cells that passed the transwell chamber were stained with crystal violet and examined using a light microscope. Quantitative analysis of the migrated number of cells in the transwell assay, on the right. ECM degradation enzymes (MMP-2, MMP-3, MMP-9, and MMP-13) were determined using western blotting in HeLa (**C**) and SiHa (**D**) cells. The protein levels of EMT molecular markers (N-cadherin, β-catenin, and vimentin) and ECM degradation enzymes (MMP-2 and MMP-9) were determined using western blotting in HeLa (**E**) and SiHa (**F**) cells. Here cropped blots were displayed and all full-length blots are included in the Supplementary Information file. *P < 0.05, **P < 0.01, ***P < 0.001, vs control.

To examine the effects of AL on migration and invasion of cervical cancer cells, we treated HeLa cells with AL and determined the migration and invasion ability using ‘wound healing’ and transwell assays. The results of the wound healing assay showed that treatment with AL resulted in a decrease in the width of scratches among HeLa cell layers in a dose- and time-dependent manner (Fig. 2A). The results of transwell assay showed that treatment with AL resulted in a decrease in the number of HeLa cells on the lower and upper chambers of the filters (Fig. 2B). Epithelial-mesenchymal transition (EMT) is closely related to the occurrence, invasion, and metastasis of tumors. To examine the effects of AL on EMT and ECM degradation enzymes of cervical cancer cells, we treated HeLa and SiHa cells with AL and determined the expression of EMT molecular markers (N-cadherin, β-catenin, and vimentin) and ECM degradation enzymes MMP-2, MMP-3, MMP-9, and MMP-13, which indicate invasion potential of cervical cancer cells. The results showed that treatment with AL resulted in a decrease in the N-Cadherin, Vimentin, MMP-3, and MMP-9 protein levels and MMP-2, MMP-3, MMP-9, and MMP-13 mRNA levels in HeLa cells in a dose-dependent manner (Fig. 2C,E). AL (5 μM) had the same effects in SiHa cells (Fig. 2D,F).

### AL promotes mitochondrial damage, induces autophagy and inhibits BMI-1

**Figure 3:**
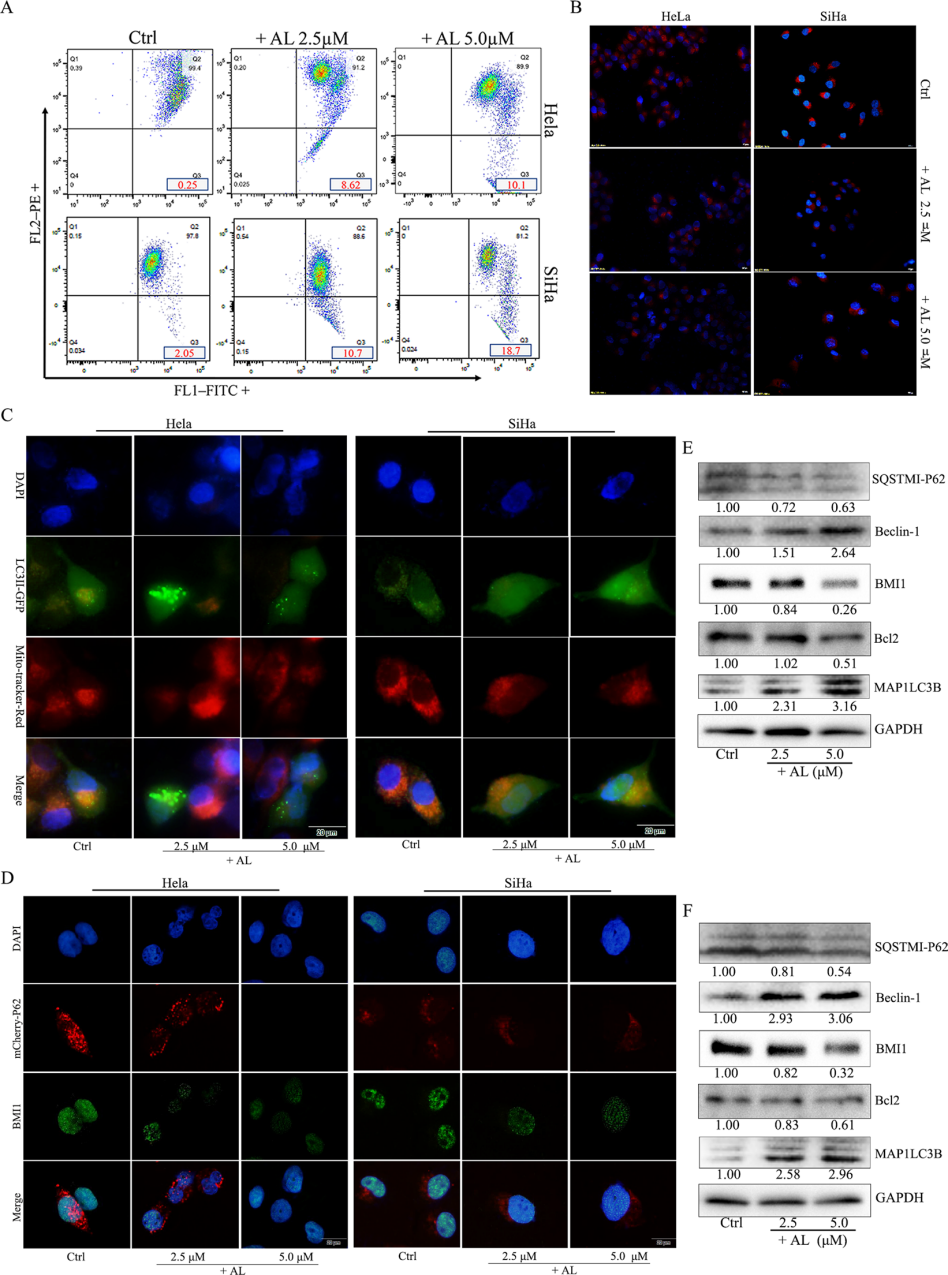
AL promoted mitochondrial damage and induced autophagy, while inhibiting BMI1 proteins in human cervical cancer cells. (**A**) HeLa and SiHa cells treated with AL (2.5, 5.0 µM) or vehicle for 24 h were stained with JC-1, and then measured using flow cytometry. (**B**) Mito-Tracker staining was used to visualize mitochondrial activity in HeLa and SiHa cells. Scale bar: 50 μm. (**C**) HeLa cells and SiHa cells were infected with Ad-GFP-LC3B for 24 h, then exposed to various concentrations of AL (0, 5, and 10 μM) for 24 h. On treatment, mitochondria labeled with Mito-Tracker Red CMXRos, and DAPI staining was used to visualize nuclei (blue). Scale bar: 20 μm. (**D**) HeLa and SiHa cells were infected with Ad-mcherry-P62 for 24 h, then exposed to various concentrations of AL (0, 5, and 10 μM) for 24 h. After the treatment, BMI1 was determined using immunocytochemistry and DAPI staining was used to visualize nuclei (blue). Scale bar: 20 μm. (**E**) The protein level of Beclin-1, BMI1, Bcl-2, LC3IIB, and P62 were detected using western blot in HeLa cells. (**F**) The protein level of Beclin-1, BMI1, Bcl-2, LC3IIB, and P62 were detected using western blot in SiHa cells. Here cropped blots were displayed and all full-length blots are included in the Supplementary Information file.

HeLa and SiHa cells were treated with different concentrations of AL for 24 h, stained with JC-1, and then measured by flow cytometry and fluorescence microscope. JC-1 exists in the form of polymer in the mitochondria of normal cells, revealing bright red fluorescence, while the green fluorescence in cells is very weak. On decreasing the mitochondrial membrane potential by AL treatment, JC-1 could not be stored in the mitochondrial matrix in the form of a polymer. At this time, the red fluorescence intensity in the mitochondria was significantly reduced, while the green fluorescence in the cytoplasm was enhanced (Fig. S2A,B). Flow cytometry detection revealed that the percentage of green fluorescent cells increased significantly (Fig. 3A). Mito-Tracker staining was used to visualize mitochondrial activity in HeLa and SiHa cells. DAPI staining was used to visualize nuclei (blue). The number of normally functioning mitochondria decreased significantly when treated with AL in a dose-dependent manner (Fig. 3B).

Autophagy is a form of programmed cell death, which is different from cell necrosis, pyrolysis and apoptosis in terms of formation mechanism and morphology. Autophagy is distinguished by the localization of the microtubule-associated protein 1 light chain 3 (LC3) protein in the autophagosome membranes in the cytoplasm and cell organelles. The activation of polyadenosine diphosphate ribose polymerase (PAPR) and caspases are rarely found in autophagy. We detected the autophagosomes labeled with Ad-GFP-LC3B and mitochondria labeled with Mito-Tracker Red CMXRos increased the number of autophagosomes, with decrease in mitochondria (Fig. 3C), which confirmed that autophagy was activated by AL-mediated mitochondrial damage. We detected the autophagy protein P62 labeled with Ad-mCherry-P62 to confirm that autophagy was activated by AL-inhibiting BMI1. The expression of BMI1 was determined using immunocytochemistry. Also, AL reduced the expression of P62 and BMI1 in a dose-dependent manner (Fig. 3D). Western blot analysis showed that AL increased the level of Beclin-1 and LC3IIB, and decreased the level of Bcl-2, P62, and BMI1 (Fig. 3E,F).

### Role of decreased BMI1 expression in inducing autophagy in cervical cancer cells and inhibiting EMT

**Figure 4:**
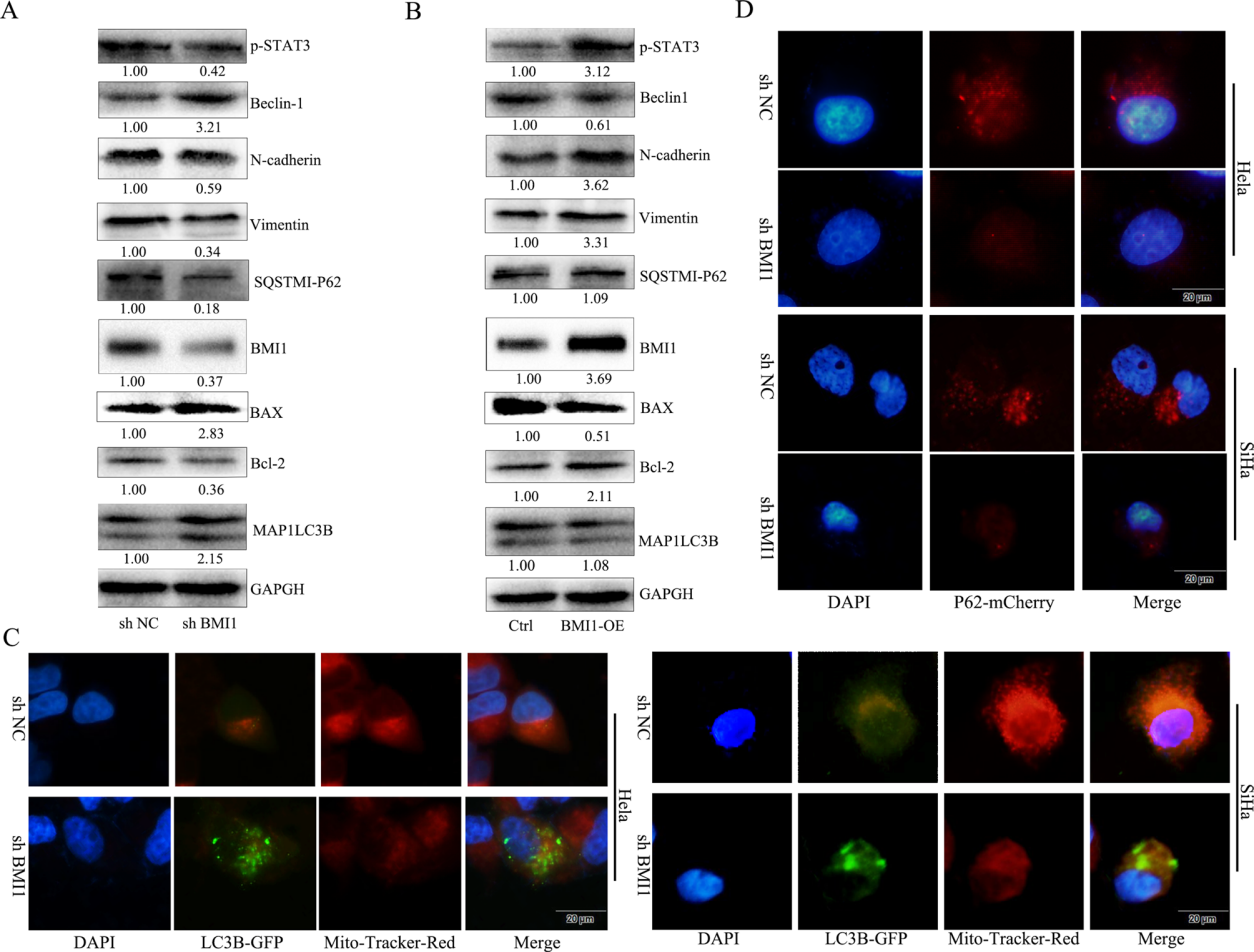
BMI1 gene expression negatively correlated with autophagy of cervical cancer cells while it positively correlated with EMT. (**A**) HeLa cells were seeded to 6-well plates at a density of 5 × 105 cells/well, transfected with shBMI1 plasmid, and protein was extracted 48 h later. BMI1, autophagy (Beclin-1, LC3IIB, Bcl-2, and P62) and EMT (N-cadherin, β-catenin, and vimentin) marker proteins as well as P-STAT3 signaling pathway were detected by western blot. (**B**) HeLa cells were seeded to 6-well plates at a density 5 × 105 cells/well, and protein was extracted 48 h later. BMI1, autophagy (Beclin-1, LC3IIB, Bcl-2, and P62) and EMT (N-cadherin, β-catenin, and vimentin) marker proteins as well as P-STAT3 signaling pathway were detected by western blot. Cropped blots were displayed in A and B, all full-length blots are included in the Supplementary Information file. (**C**) HeLa and SiHa cells were seeded to 24-well plates at a density 5 × 10^5^ cells/well, transfected with shBMI1 plasmid for 24 h, infected with Ad-GFP-LC3B for 24 h, and then mitochondria were stained with Mito-Tracker Red CMXRos. Fluorescence microscope was used to observe the increase in autophagosomes and mitochondrial damage. Scale bar: 20 μm. (**D**) HeLa and SiHa cells were seeded to 24-well plates at a density of 5 × 10^5^ cells/well, transfected with shBMI1 plasmid for 24 h, infected with Ad-mcherry-P62 for 24 h, and then fluorescence microscope was used to observe expression of autophagy-related protein P62. Scale bar: 20 μm.*P < 0.05, vs control.

BMI1 is associated with self-renewal of cancer stem cells and sensitivity of tumors to chemoradiotherapy. Reducing expression of BMI1 promotes apoptosis and/or aging of tumor cells, while enhancing sensitivity of tumor cells to chemoradiotherapy^21^. Inhibiting expression of BMI1 can promote autophagic death in tumor cells^20^. To verify the effects of BMI1 in cervical cancer cells and analyze the relationship between this gene and AL-induced autophagy, BMI1 knockdown hairpin plasmid and overexpression plasmid were designed and used to transfect HeLa cells for 24 h. Then, western blot was used to detect BMI1, autophagy (Beclin-1, LC3IIB, Bcl-2, and P62) and EMT (N-cadherin, β-catenin, and vimentin) marker proteins as well as P-STAT3 signaling pathway. The results showed that both knockdown and overexpression of BMI1 gene were successful (Fig. S3). Down-regulation of BMI1 expression promoted high expression of autophagy-related genes Beclin-1 and LC3IIB, decreased the expression of substrate P62 of Bcl-2 and LC3IIB, decreased the expression of EMT-related proteins, and inhibited P-STAT3 signaling pathway (Fig. 4A). Overexpression of BMI1 showed that indicators were reversed (Fig. 4B). To detect the induction of autophagy by decreasing BMI1 expression and its association with mitochondrial damage, BMI1 gene was knocked down, the HeLa and SiHa cells were infected with Ad-GFP-LC3B for 24 h, and then mitochondria were stained with Mito-Tracker Red CMXRos. The results showed that BMI1 gene knockdown induced autophagosome formation accompanied by a reduction in mitochondria (Fig. 4C). After the expression of BMI1 gene was suppressed, HeLa and SiHa cells were infected with Ad-mcherry-P62 for 48 h, and fluorescence microscopy was used to detect the expression of P62. It was found that BMI1 gene knockdown induced a decrease in P62 expression, indicating that the occurrence of autophagy negatively correlated with BMI1 expression (Fig. 4D).

### AL suppressed the tumorigenicity of HeLa cells in vivo

**Figure 5:**
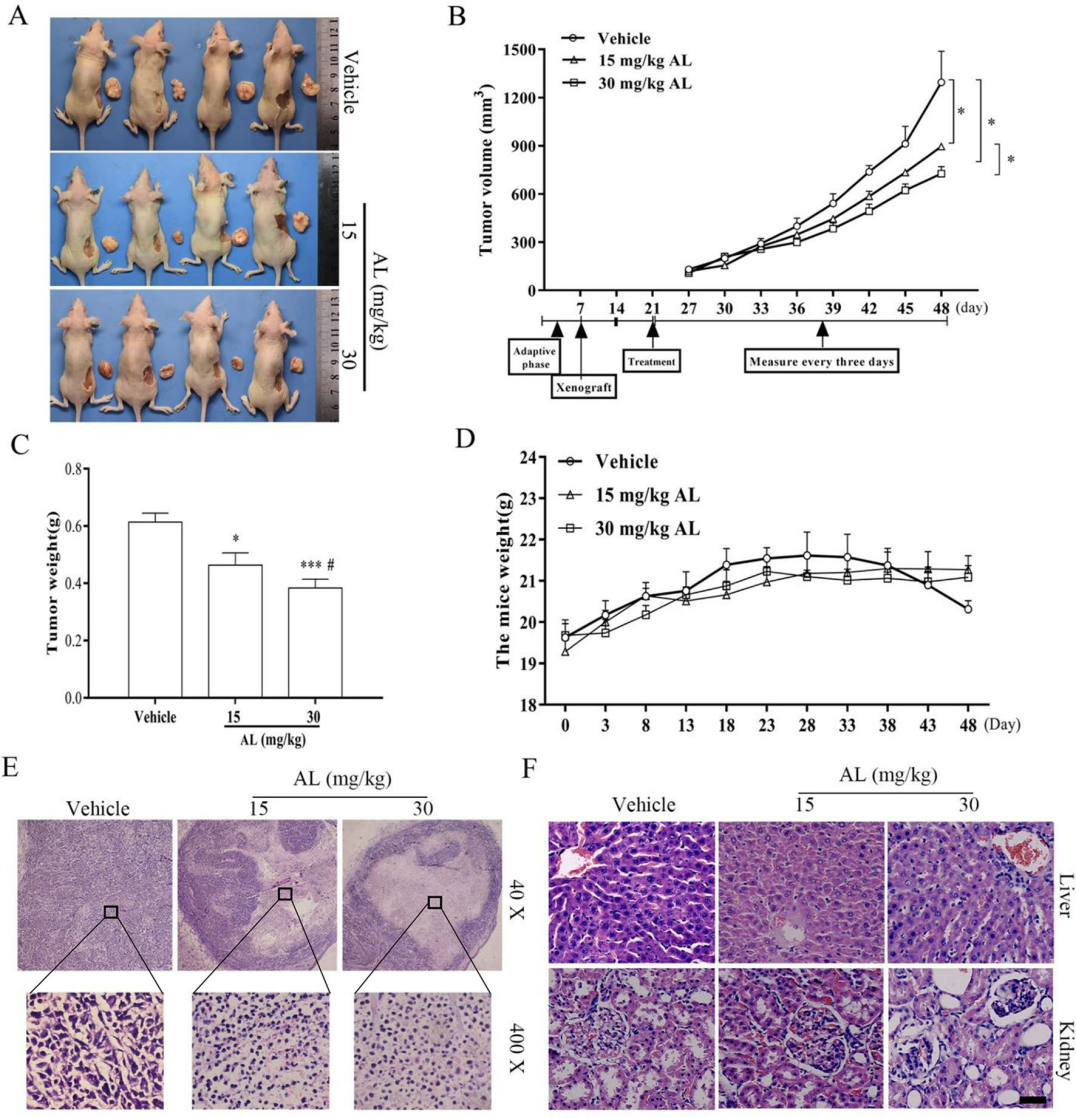
AL inhibited the growth of mouse cervical cancer xenografts model established with HeLa cells. (**A**, **B**) Tumor volumes, (**C**) tumor weights and (**D**) body weights of tumor-bearing nude mice in AL group and control groups. *P < 0.05, ***P < 0.001, vs vehicle group. ^#^*P* < 0.05, vs AL 1 5 mg/kg group. (**E**) Morphology of tumor xenograft from each mouse. (**F**) H&E staining of main organs of tumor-bearing nude mice after treatment. Scale bar: 25 μm.

To examine the effects of AL on the growth of cervical cancer tissues in vivo, we treated a Balb/C female nude mice model of cervical cancer derived from HeLa cells with AL or saline, and examined the tumor tissues, and measured the weight and volume of tumor mass. The results showed that the weight (Fig. 5C) and volume (Fig. 5A,B) of tumors in the AL treatment group were significantly less than those of the saline treatment group. The abscissa in Fig. 5B shows the process of the animal experiment. The body weight of the experimental mice did not change significantly during the treatment (Fig. 5D). At the same time, H&E detection showed that necrosis occurred in tumor tissues of the AL group (Fig. 5E), whereas the morphology of liver and kidney tissues was normal and AL did not exhibit toxicity to liver and kidney tissues (Fig. 5F). The results suggest that AL significantly inhibited the growth of cervical cancer cells in dose-dependent manner in nude mice.

### Effect of AL induces autophagy and inhibits epithelial-mesenchymal transition by inhibiting BMI1 protein in vivo

**Figure 6:**
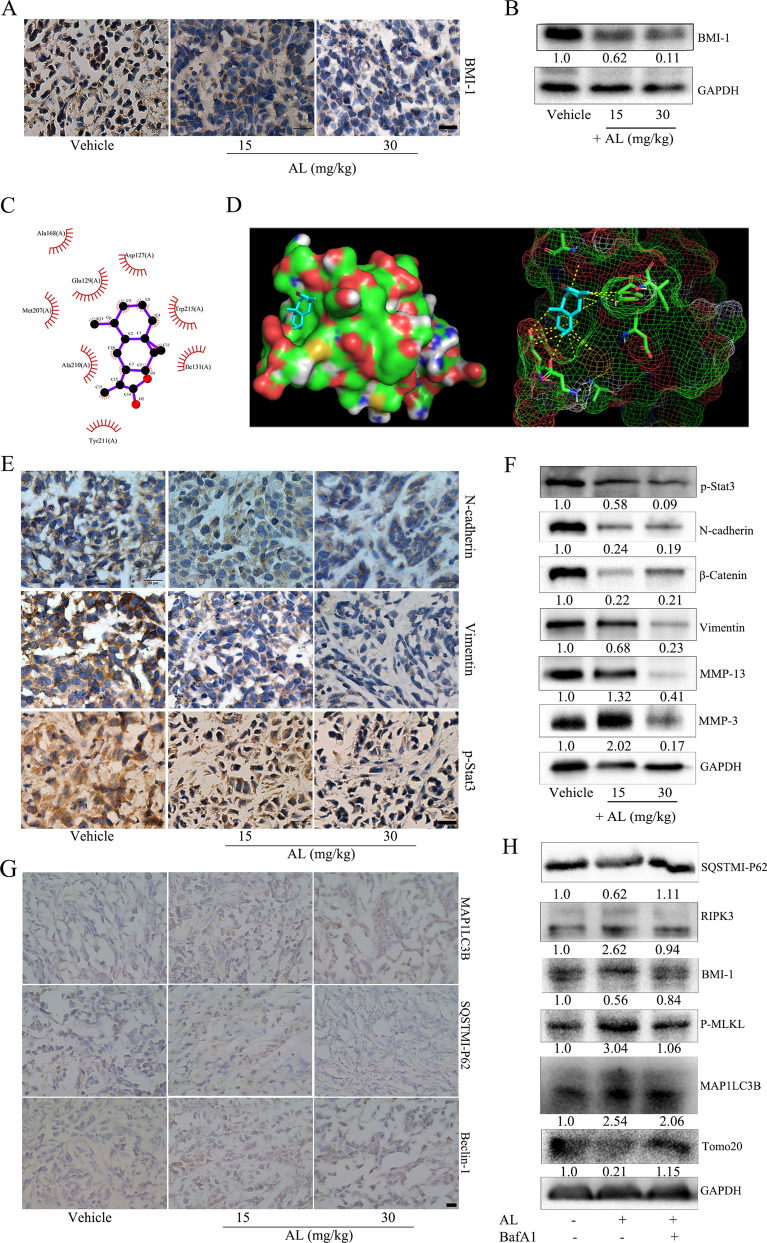
AL induces autophagy and inhibits epithelial-mesenchymal transition by inhibiting BMI1. (**A**) BMI1 protein levels in the tumor tissues grown from HeLa cells were determined using immunohistochemistry. Scale bar: 20 μm. (**B**) BMI1 protein levels in the tumor tissues grown from HeLa cells were determined using western blot. (**C**) Molecular docking between AL and BMI-1. 2D ligand-receptor interaction representation. The interaction between alantolactone and BMI1 predicts hydrophobic contacts and was drawn with LigPlus version 2.1 (**D**) 3D schematic representation of interactions between alantolactone and BMI1 5FR6. Molecular docking was performed by AutoDock Vina and visualized using Pymol 2.3. Any interaction below 3 Å between alantolactone and BMI1 was presented by Pymol 2.3. (**E**) p-STAT3Tyr705, N-cadherin, and vimentin protein levels in the tumor tissues grown from HeLa cells were determined through immunohistochemistry. Scale bar: 20 μm. (**F**) p-STAT3 (Tyr705), BMI1, N-cadherin, β-catenin, vimentin, MMP-3, and MMP-9 protein levels in the tumor tissues grown from HeLa cells were determined through western blot. (**G**) P62, LC3B, and Beclin-1 were determined through immunohistochemistry. Scale bar: 20 μm. (**H**) P62, BMI1, P-MLKL, LC3B, and Tomo20 were determined through western blot after HeLa cells were treated with AL or AL and autophagy inhibitor bafilomycin A1. Here cropped blots were displayed and all full-length blots are included in the Supplementary Information file.

First, we found that AL could also inhibit BMI-1 expression in vivo (Fig. 6A,B) using immunohistochemistry and western blot. In silico molecular docking revealed that alantolactone was predicted to have a stronger binding affinity with 5FR6 (− 6.1 kcal/mol) than 3RPG (− 5.9 kcal/mol). The strongest binding site between alantolactone and BMI1 is shown in Fig. 6C,D. Therefore, we assume that AL inhibited EMT in cervical cancer cells to promote autophagy by inhibiting BMI-1 expression. Further detection revealed that the expressions of proteins related to EMT and extracellular matrix degradation were also inhibited (Fig. 6E,F). The IHC (Fig. 6G) results showed that the protein level of Beclin-1 and LC3B increased, whereas the protein level of P62 decreased in the tumor tissues grown from HeLa cells, in the AL treatment groups compared with the control groups. We used autophagy inhibitor Ba to verify that AL promoted autophagic cell death by inhibiting BMI-1 expression (Fig. 6H).

## Discussion

High expression of BMI1 is significantly associated with poor tumor differentiation, high clinical grade, lymph node metastasis, and poor prognosis of cervical cancer^22–24^, and is an independent prognostic factor in cervical carcinoma^23–25^. Aberrantly elevated BMI1 promotes cervical tumorigenicity and tumor sphere formation via enhanced transcriptional regulation of Sox2 genes as a potential oncogenic factor that participates in the carcinogenesis of cervical carcinomas^22^. The increase in the ability of tumor cells to form spheres indicates that stem cells or mesenchymal level is enhanced, and the BMI1 gene plays an important role in maintaining self-renewal of tumor stem cells^21,26,27^ and has a pivotal role in chemotherapy sensitivity, tumor resistance, and tumor recurrence. Epithelial-mesenchymal transition (EMT) is a key developmental process that is often activated during tumorigenesis^28,29^. EMT is related to cell stemness^30,31^. BMI1 and TWIST1 function together to promote tumor dedifferentiation, metastasis, and development of drug resistance^32^. As a potential anti-inflammatory target, BMI1 regulates invasion and EMT of colorectal cancer cells via TLR4/MD-2 MyD88 complex-mediated NF-κB signaling pathway^33^.

Evidence from in vitro studies demonstrated that the main absorption mechanism of AL was passive diffusion and demonstrated good intestinal absorption^34^. The study showed poor absorption of AL in vivo^35^*.* High body clearance and low oral bioavailability, the highest concentration was achieved in the small intestine and feces clearance was shown to be the dominant elimination pathway of the lactones^36^. But AL has a rapid onset and does not cause significant damage to normal animal tissues and organs^35–37^. AL possesses multiple pharmacological activities, and its anti-tumor activity is highly attractive^38–40^. However, the pivotal molecules targeted by AL remain unclear. AL induces apoptosis and cell cycle arrest with low cytotoxicity and exerts an inhibitory effect on tumor cells^41^. AL selectively inhibits activation of STAT3 and exhibits potent anticancer activity in MDA-MB-231 cells^42^. By inhibiting NF-kB and its downstream target proteins, AL induces apoptosis in a dose-dependent manner and selectively ablates acute myeloid leukemia stem and progenitor cells^43^. Isoalantolactone, a derivative of AL, induces apoptosis of human breast cancer cells through ROS-mediated mitochondrial pathway and down-regulation of SIRT1 expression^44,45^. Studies have confirmed that AL also exerts a good inhibitory effect on cervical cancer cells. AL exhibits selective antitumor effects in human HeLa cervical cancer cells by inhibiting cell migration and invasion, G2/M cell cycle arrest, mitochondrial-mediated apoptosis, and targeting of NF-kB signaling pathway^46^. AL induces apoptosis of cervical cancer cells through generation of reactive oxygen species, glutathione depletion, and inhibition of Bcl-2/Bax signaling pathway^38^. However, the effects of AL on cervical cancer in vivo have not been reported, nor has the induction of autophagic death by AL in cervical cancer cells.

Does AL exert its inhibitory effects on cervical cancer through BMI1/SATA3 signaling axis? Our study indicated that AL could significantly inhibit growth of cervical cancer cells, promote apoptosis (Fig. 1), inhibit cell migration and invasion (Fig. 2), and significantly inhibit expression of genes associated with EMT (N-cadherin, vimentin, and β-catenin) and extracellular matrix degradation (MMP-2, MMP-9, MMP-3, and MMP-13). The results of Caspase-3 activity detection and apoptosis detection with flow cytometry showed that AL did not activate Caspase-3 when promoting apoptosis in HeLa and SiHa cells (Fig.S1). However, the effects of AL on cell mitochondrial membrane potential were obvious (Figs. S2 and 3A), and the number of mitochondria also decreased, as the concentration of AL increased (Fig. 3B).

Does AL exert its inhibitory effects on cervical cancer cells by inducing autophagic cell death? The detection of autophagosomes and mitochondria showed that with the increase of AL concentration, the number of autophagosomes increased significantly while that of mitochondria decreased significantly in HeLa cells. Western blot detection showed that AL significantly promoted upregulation of expression of autophagy-related proteins Beclin-1 and LC3B and downregulation of P62 expression, inhibited expression of BMI1 protein (Fig. 3C–F). Knocking down BMI1 gene increased the number of autophagosomes and decreased the number of mitochondria (Fig. 4C), while significantly up-regulating expressions of autophagy-related genes Beclin-1 and LC3B, down-regulating P62 expression, and down-regulating expressions of EMT-related proteins. By BMI1 gene overexpression, the detection of the above indicators showed opposite results (Fig. 4A,B,D). It should be noted that the expression level of p-STAT3^Tyr705^ by BMI1 gene knockdown significantly decreased, and that of p-STAT3^Tyr705^ by BMI1 gene overexpression significantly increased (Fig. 4A,B). It showed that BMI1 gene plays an important role in cervical cancer progression and closely correlates with autophagy of cervical cancer cells. AL may target BMI1 to induce autophagic cell death and exert potent inhibitory effects on cervical cancer. Through molecular docking simulation, it was found that AL could directly bind with BMI1 (Fig. 6C,D). To further verify the anti-tumor effects of AL in vivo, we established an animal model of cervical cancer with subcutaneous implantation of HeLa cell tumor in nude mice, with intragastric administration in the two dose groups, to detect inhibitory effects of AL on cervical cancer cells in vivo. Wang, et al. used 10 mg/kg and 20 mg/kg AL against glioblastoma by daily intraperitoneal injections^37^. Liu et al. also performed an in vivo study of breast cancer by intraperitoneal injection of 5 mg/kg of AL^39^. Wang, et al. assessed the therapeutic effect of the AL on prostatic cancer in vivo with 50 mg/kg AL once daily by oral gavage^37^. Yin, et al. assessed AL inhibits tumor growth of TNBC in vivo with 15 mg/kg and 30 mg/kg AL once daily by oral gavage^40^. Studies in rat reported a plasma C_max_ of 1.103 mg/L (4.75 μM) after intravenous administration of Radix Inulae extract containing 3.43 mg/kg AL^47^ and 0.03 mg/L (0.12 μM) after oral administration at a dose of 50 mg/kg AL^36^. Considering the pharmacokinetic characteristics and administration mode of AL, 15 mg/kg and 30 mg/kg of AL were used to treat subcutaneous xenograft tumors of HeLa cells in this study. The results revealed that AL exerted significant inhibitory effects on cervical cancer cells in vivo in a dose-dependent manner (Fig. 5A–C). Pathology section examination showed that AL group tumor tissues exhibited necrosis (Fig. 5E) whereas there was no damage to internal organs (Fig. 5F), indicating that AL is an effective cervical cancer inhibitor with low toxicity. Immunohistochemical results of tumor tissues also verified the inhibitory effects of AL on BMI1 in cervical cancer tissues (Fig. 6A,B), and its regulating effects on EMT (Fig. 6E,F) and autophagy (Fig. 6G). Previous studies have confirmed that necrosis is a new mode of caspase-independent cell death that is different from apoptosis. It relies on activation of RIPK1 and RIPK3^48^. Once active, RIPK3 then phosphorylates MLKL, which is the final stage of necrosis^49^. In ovarian cancer cells, inhibition of BMI1 gene can induce activation of RIPK1-RIPK3 complex, which phosphorylates its downstream substrate MLKL and enhances necrosis^20^. Our results confirmed that AL promoted upregulation of RIPK3 expression in cervical cancer HeLa cells and enhanced phosphorylation level of MLKL, which was reversed in the presence of bafilomycin A1 (BafA1) (Fig. 6H). BafA1 inhibited the binding of autophagosomes to lysosomes. Therefore, BafA1 had no effects on LC3B expression but inhibited P62 degradation. It showed that AL could induce autophagic death in cervical cancer cells by inhibiting BMI1 expression.

**Figure 7:**
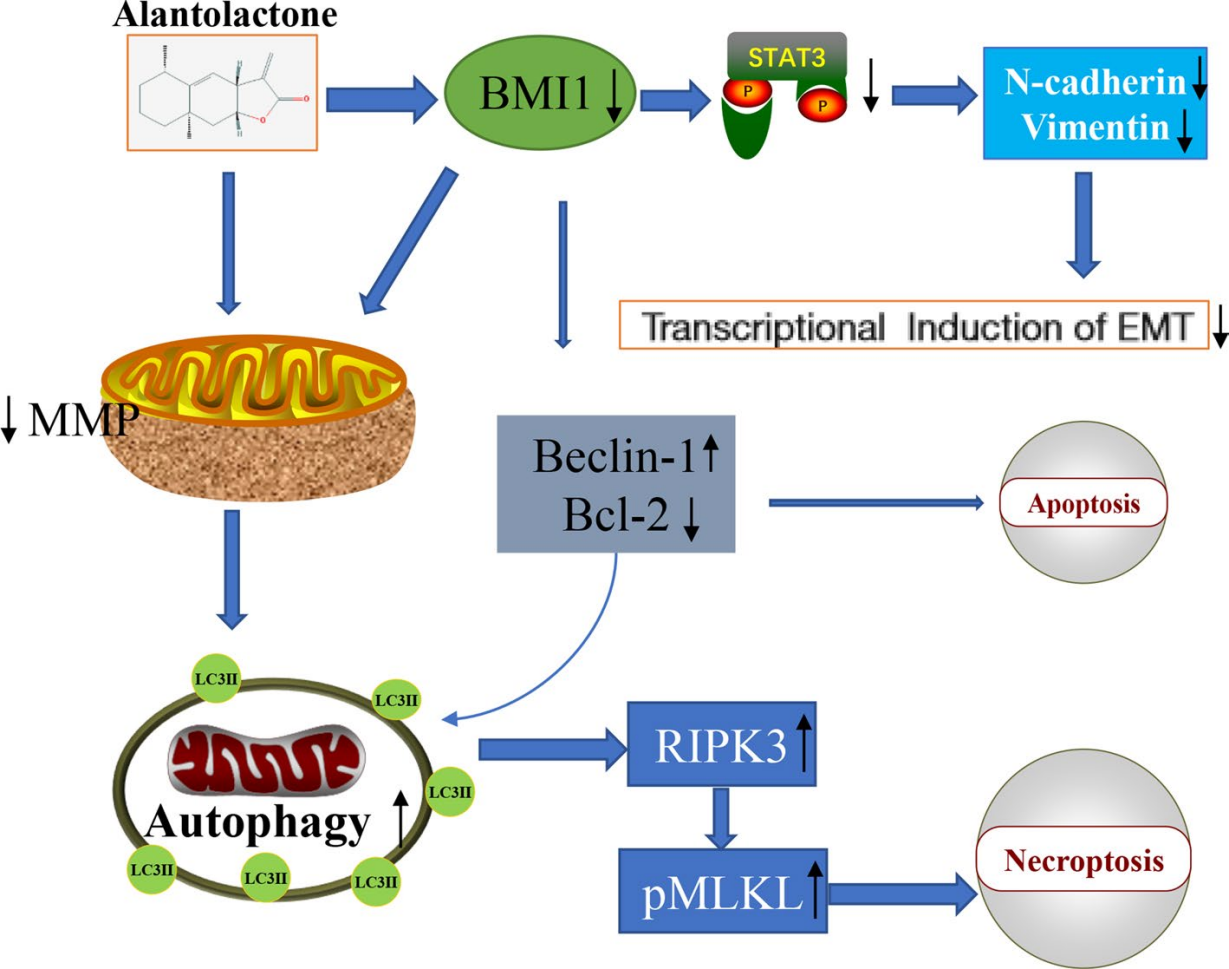
Schematic diagram of the mechanisms contributing to AL inhibiting cervical cancer progression. AL causes depletion of BMI1 to induce autophagy-dependent necroptotic cell death and inhibits epithelial-mesenchymal transformation through the STAT3 signaling pathway in cervical cancer cells.

In summary, the inhibitory effect of AL on cervical cancer may be based on multiple factors. It was observed that AL has a significant effect on BMI1 inhibition, and BMI1 positively promotes the proliferation and metastasis of tumor cells. This may be one of the anti-tumor mechanisms of AL (Fig. 7). Therefore, AL is a potential natural chemotherapeutic compound for treating cervical cancer and its anti-tumor mechanism deserves further investigation.

## Methods and materials

### Chemicals and reagents

AL (purity > 98%) was purchased from Tauto Biotech (Shanghai, China). AL was dissolved in DMSO and the final concentration of DMSO in cell culture was kept below 0.05%. Dulbecco’s phosphate buffered saline (DPBS) was purchased from Sigma Aldrich (St. Louis, MO). Cell Counting Kit-8 (CCK-8) kit (BS350B) was from Biosharp (Hefei, China). Matrigel was purchased from BD Biosciences (SanJose, CA, USA). The primary antibodies for BMI1, BCL-2, BAX, Beclin-1, P62, LC3II, MMP-3, MMP-9, N-cadherin, and Vimentin were from Abcam (Cambridge, MA, UK). Primary antibodies for p-STAT3(Tyr705), STAT3, and GAPDH and all secondary antibodies were from Cell Signaling Technology (Beverly, MA, USA). Penicillin, streptomycin, DMEM (high glucose), RPMI1640 medium, and fetal bovine serum (FBS) were obtained from GIBCO BRL (Grand Island, NY, USA). Trizol reagent (93289-100ML) was from Sigma-Aldrich (St. Louis, MO, USA), reverse transcription kit (M1705) was from Promega (Madison, WI, USA), and SYBR Green PCR Mix (1725204) was from Bio-Rad (Hercules, CA, USA). Annexin V-FITC/propidium iodide apoptosis detection kit was from BD Biosciences (San Jose, CA, USA). PI/RNase Staining Buffer Kit was from KeyGEN (Suzhou, China). Ad-GFP-LC3B (C3006-1 ml), Ad-mCherry-P62 (C3016-1 ml), Ad-mCherry-GFP-LC3B (C3011-1 ml), and Mito-Tracker Red CMXRos (C1049-50 µg) were from Beyotime (Hangzhou, China). Applied Biosystem 7500 real-time PCR machine was from Thermo Fisher Scientific (Waltham, MA, USA). SpectraMax iD3 spectrophotometer was from Molecular Devices (San Jose, CA, USA). BD FACSAria II flow cytometry was from BD Biosciences (San Jose, CA, USA). IX73 inverted fluorescence microscope was from Olympus (Tokyo, Japan). Microplate reader and Nanodrop 2000 were from Thermo Fisher Scientific (Waltham, MA, USA).

### Cell culture

Human cervical carcinoma cell lines HeLa cells (HPV18 positive)^50^ and SiHa cells (HPV16 positive)^51–53^ were purchased from Hunan Feng Hui Biological Technology Co., Ltd. (Hunan, China). These cells were certified by STR test. HeLa and SiHa cells were cultured with DMEM (Gibco, 11965-092) containing 10% fetal bovine serum (FBS, Capricorn, FBS-HI-11A) and 100 IU/ml of penicillin G sodium and 100 mg/ml of streptomycin sulfate at 37 °C in an incubator with 5% CO_2_/95% humidified air. Cells in exponential growth phase were used for the experiment.

### Determination of mitochondrial membrane potential using fluorescence microscope and flow cytometry

Cell mitochondrial membrane potential was measured using the Mitochondrial Membrane Potential Assay Kit (KeyGEN, China). JC-1 is a cationic lipid fluorescent dye that can selectively enter mitochondria. JC-1 forms a multimer in normal mitochondria and emits red fluorescence. When the mitochondrial transmembrane potential is depolarized, JC-1 is released from the mitochondria to the cytoplasm, the intensity of red light is weakened, and emits a green fluorescence. The cells were stained with JC-1 for 15 min after intervention, following the manufacturer's instructions. Fluorescence was determined by using the fluorescence microscope and flow cytometry microscopy.

### Mito-tracker red CMXRos staining

Cells were stained with Mito-Tracker Red CMXRos dye (Beyotime, China) and DAPI (4′, 6-diamidino-2-phenylindole) (Beyotime, China). Corresponding treated cells were incubated with Mito-Tracker Red CMXRos for 30 min at 37 °C. After dyeing, 4% polyformaldehyde was used to fix the cells for 30 min at room temperature. On fixing, cells were incubated with DAPI for 5 min at room temperature. The fluorescence of the stained cells was determined by using the fluorescence microscope.

### Cell immunofluorescence assays

5 × 10^3^ Cells were seeded on 24-well plates and treated with AL for 48 h. Fixed with 4% paraformaldehyde at room temperature for 30 min, cells were permeabilized with 0.1% triton X-100 for 30 min, blocked with 5% BAS for 1 h, and incubated overnight with BMI1 antibodies at a dilution of 1:200. Dylight549-conjugated goat anti-rabbit IgG and Dylight488-conjugated goat anti-rabbit were used as the secondary antibody. The nuclei were counterstained with 4′,6-diamidino-2-phenylindole (DAPI). The method cited the references^54^. Images were captured using IX73 inverted fluorescence microscope.

### Mice tumor models

Eighteen female athymic BALB/C nude mice weighing 14–20 g (4 weeks) were purchased from Hunan SJA Laboratory Animal Co., Ltd. (Changsha, China). The mice were housed at 20 °C–22 °C with 50%–60% relative humidity and fed with standard laboratory chow and tap water ad libitum. 5 × 10^6^ HeLa cells were suspended in 100 µl PBS and subcutaneously injected into the right armpit of all BALB/C mice. When the tumors were approximately 100 mm^3^ in size, mice were randomly assigned to three groups, the control group administered vehicle (normal saline containing 0.1% ethanol, 3 times/week, intragastric administration), the AL-low dose group administered AL (15 mg/kg, 3 times/week, intragastric administration), and the AL-high dose group administered AL (30 mg/kg, 3 times/week, intragastric administration). The dose of AL was referred to the report^40^. The treatment lasted for 4 weeks. The key time nodes are explained in Fig. 6B. Food, water intake, and behavioral changes were monitored daily, the body weight and tumor volumes were recorded every 3 days throughout the test period. Tumor volumes were calculated with the tumor length and width, which were measured using a caliper: tumor volume = (length) × (width)^2^  × 0.5. Subcutaneous xenotransplantation of HeLa cells and tumor measurement methods were reference to relevant literature^55^. At the end of the treatment, all the mice were sacrificed by cervical dislocation. Tumors were isolated, weighed, and aliquoted for western blot analysis, hematoxylin–eosin (H&E) staining, and immunohistochemical (IHC) staining assay. Mouse xenograft experiments in this study complied with the ARRIVE guidelines and were conducted in accordance with the U.K. Animals (Scientific Procedures) Act, 1986 and associated guidelines. This study was approved by the Ethical Committee for Animal Experimentation of Xiangyang No.1 People’s Hospital (NO. 2018DW016).

### Immunohistochemistry staining of cancer tissues

Tumor tissues were fixed in 4% polyformaldehyde, and paraffin-embedded and sectioned into 5 μm slices. The expression of P-STAT3, BMI1, N-cadherin, Vimentin, MMP-3, MMP-9, LC3B, and P62 in tumor tissues was examined using immunohistochemistry staining. Brown-yellow staining was considered positive. The stained section was examined under a microscope and four fields were selected in the areas with clear cell staining and good background. The view was recorded in images and analyzed using Image J software (National Institutes of Health). The relative expression of P-STAT3, BMI1, N-cadherin, Vimentin, MMP-3, MMP-9, LC3B, and P62 was calculated based on the positively stained area and the total area in the views.

### Molecular docking

We performed in silico prediction of BMI1 and alantolactone binding affinity by using AutoDock Vina (version 1.1.2)^56^. The 3D and 2D schematic visualization of binding site was generated by PyMol (version 2.3)^57^ and LigPlus (version 2.1)^58^ separately. The structure pdb file of BMI1 (3RPG and 5FR6) was extracted from The Research Collaboratory for Structural Bioinformatics Protein Data Bank database^59,60^ (http://www.rcsb.org/). 3RPG consist of 1–109 amino acids of BMI1 and 5FR6 consist of 121–235 amino acids of BMI1. These two molecules represent the whole structure of BMI1. We obtained alantolactone structure (CID:72724) from the PubChem database^61^. The ligand and receptors were processed using vina tutorial in the AutoDock Tools^62^. As this was blind docking, we set the grid box to be large enough to surround the whole molecule.

Partial experimental methods are elaborated in supplementary materials 1.

### Statistical analysis

All data were analyzed using GraphPad Prism 7.0 (GraphPad Software, San Diego, CA) software and presented as the mean ± standard deviation. Values represent the mean ± SD from one representative experiment of three independent experiments, each performed in triplicate. Data was analyzed using the unpaired Student’s t-test. Differences among test groups were analyzed by ANOVA. *P* values < 0.05 were considered statistically significant.

### Ethical approval

Mouse xenograft experiments in this study complied with the ARRIVE guidelines and were conducted in accordance with the U.K. Animals (Scientific Procedures) Act, 1986 and associated guidelines. This study was approved by the Ethical Committee for Animal Experimentation of Xiangyang No. 1 People’s Hospital (NO. 2018DW016).

## Supplementary Information

Supplementary Information.
