# Supplementary material for: Alantolactone inhibits cervical cancer progression by downregulating BMI1
Source: Sci Rep. 2021 Apr 29;11:9251. doi: 10.1038/s41598-021-87781-z (PMC8085045; doi:10.1038/s41598-021-87781-z)
Supplement: Supplementary file 1 — Supplementary Information. [file 41598_2021_87781_MOESM1_ESM.docx]

**Alantolactone Inhibits Cervical Cancer Progression by Downregulating BMI1**

Xiaodong Sun**^1Δ^**, Hongxia Xu**^1Δ^**, Tianyu Dai^2^, Lixia Xie**^1^**, Qiang Zhao **^1^**, Xincai Hao^1^, Yan Sun^1^, Xuanbin Wang^1^, Nan Jiang^3^, Ming Sang**^1^***

^1^ Hubei Institute of Parkinson's Disease at Xiangyang No.1 People’s Hospital, Hubei Key Laboratory of Wudang Local Chinese Medicine Research, Hubei University of Medicine, Shiyan 442000, People’s Republic of China.

^2^ Department of Joint Surgery, Shanghai East Hospital, School of Medicine, Tongji University, Shanghai 200092, People’s Republic of China.

^3^Hubei Province Hospital of Traditional Chinese Medicine, Hubei Province Academy of Traditional Chinese Medicine, Wuhan, 430061, People’s Republic of China.

* **Corresponding author**: Dr. Ming Sang, Hubei Institute of Parkinson's Disease at Xiangyang No.1 People’s Hospital, Hubei Key Laboratory of Wudang Local Chinese Medicine Research, Hubei University of Medicine, Shiyan 442000, People’s Republic of China. Email: sangming@whu.edu.cn or smxd2000@126.com

Address: 15 Jiefang Road, Xiangyang 441000, Hubei, People’s Republic of China

Telephone number: Office, 086-710-3420011 Lab, 086-710-312400

**^Δ^**These authors are equal contributors to this work.

**Supplemental methods:**

**Cell proliferation using CCK-8 assay**

HeLa cells and SiHa cells were seeded to 96-well plates at a density of 10000 cells/well. After incubating for 30 minutes in an incubator at 37 °C cells were treated with resveratrol or control vehicle and cultured at 37 °C for 24, 48, and 72 h. Cells were washed once with 125 μL phosphate-buffered saline (PBS)/well and assayed using a CCK-8 kit according to the manufacturer’s instructions. The 96-well plates were read at 450 nm on a plate reader (SpectraMax iD3，Molecular Devices，USA). Cell viability was denoted by the percentage of cell loss, which was calculated using the formula:(1-Drug_A450_/Control_A450_) x100, where A450 denotes absorbance at a wavelength of 450 nm.

**Colony formation assay**

Suspension of individualized HeLa cells and SiHa cells was made from cultured cells by trypsin digestion and pipetting. The cell suspension was diluted with DMEM containing 10% FBS and desired concentrations of resveratrol or vehicle control and then aliquoted to 6-well plates at a density 100 cells/well. After a 14-day culture, cells were washed with cold PBS twice and fixed with 3.7% formaldehyde. After cell colonies were stained with Crystal violet (Sinopharm Chemical Re-agent Co., Shanghai, China), the numbers of colonies in each well were counted.

**Determination of cell apoptosis by flow cytometry**

HeLa cells and SiHa cells were treated with AL for 24 h and then collected by digestion with 0.25% tryptase (Gibco, 25300054). After washing with pre-cooled PBS, cells were collected by 2000 rpm centrifugation for 5 min at 4 ^o^C, resuspended with 1x Binding Buffer of an apoptosis test kit (Beijing Ambition Biotechnology Co., Ltd. ABXBIO, ABS50001), and mixed with 5 µL Annexin V-FITC. The cells were incubated at room temperature for 15 min, then stained with 5 µL PI for 5 min. After 200 µL 1 x binding buffer was added, the cells were analyzed on a flow cytometer (BD FACSAria II, BD, USA) at 488 nm.

**GreenNuc Caspase-3 Assay**

HeLa and SiHa cells were seeded to black 96-well plates at a density of 10^4^ cells/well. After treatment with AL for 24 h, cells were added with GreenNuc Caspase-3 Substrate (5 μM) and inhibitor Ac-DEVD-CHO (20 μM), and incubated at room temperature for 15–30 m. A plate reader was set with an excitation wavelength of 485 nm and an emission wavelength of 515 nm, on which fluorescence values were read. Then, a fluorescence microscope was used to detect and photograph cells, and cell nuclei were counterstained with Hoechest 33342.

**Wound healing assay**

The effects of resveratrol on migration of HeLa cells were examined using wound healing assay. HeLa cells were seeded in a 6-well plate at a density 5×10^5^ cells/well. After 80% confluence, a scratch was made across the cell monolayer on the bottom of plates with a 200 μl sterile pipette tip and washed with PBS. The cultures were then treated with resveratrol at indicated concentrations or untreated (blank control) and incubated for 0, 24, 48, and 72 h and images of the cultures were taken with an inverted microscope (IX73P2F, Olympus Optical Co., Ltd., Japan). The scratches across the cell culture were measured using Olympus cellSens software (Olympus, Japan) based on the images. The experiment was repeated three times.

**Invasion assay**

The effects of resveratrol on invasion of HeLa cells were examined using Transwell assay. Boyden chambers containing 24-well Transwell plates (Corning Inc., USA) with 8 mm pore size were used. HeLa cells were seeded at a density 1 × 10^5^ cells/ ml in the upper chambers coated with Matrigel. These cells were treated with 0, 10, 20, and 40 μM resveratrol dissolved in medium. DMEM medium containing 10% FBS was added to the bottom chamber. After culture for 24 h, the filters in the upper chambers were collected, cells on the upper side of the filter membrane were wiped out with a cotton swab and the invaded cells on the lower side of the filter membrane were fixed in 4% paraformaldehyde to the slides, followed by staining with 0.1% crystal violet for 10 min (Sinopharm Chemical Re-agent Co., Shanghai, China). The cells in the slides were examined and counted in five randomly selected microscopic fields (×400) using an inverted microscope (IX73P2F, Olympus Optical Co., Ltd., Japan). The number of cells in various treatment groups were compared. All experiments were done thrice.

**HeLa cells infected with adeno-associated viruses Ad-GFP-LC3B and Ad-mCherry-p62**

HeLa and SiHa cells were seeded to 24-well cover glasses at a density of 10^5^ cells/well. Culture medium was replaced with 800 μl fresh culture medium after the cells were cultured for 24 h and completely adherent. The experiment was conducted according to the biological safety standards of US CDC and its requirements for operations and protection. Cells were infected with adenoviruses at a MOI of 5 per well, and the culture medium containing viruses was removed about 24 h after infection. One ml of fresh complete culture medium was added to each well, and the cells were grouped and intervened. After culturing for 24 h, cell nuclei were stained with Hochest. The cell glasses were taken out and placed upside down on slides with a mounting medium after washing with PBS. Fluorescent microscope was used to observe cell growth and fluorescent protein expression, and the level of autophagy was determined according to fluorescence intensity.

**Overexpression and knockdown of BMI1 gene**

HeLa and SiHa cells at logarithmic phase were collected and the concentration of cells was adjusted to 5×10^4^/ml. Cells were seeded into 6-well plates at 2 ml/well for protein extraction, and 24-well plates on which cell glasses were placed at 500 µl/well for fluorescence photography. No-load control group, overexpression group/knockdown group were set. When cell confluence reached 60–70%, cells were transfected with plasmids, cultured in a 5% CO2 incubator at 37 °C for 24 h, and followed by continuous culture in AL with a final concentration of 5.0 µM for 24 h. BMI recombinant plasmids were synthesized by Hunan Fenghui Biotechnology Co., Ltd. and shRNA interference sequences were designed by Shanghai GenePharma Co., Ltd.

**Real-time RT-PCR**

Total RNA was extracted using TRIzol reagent (Sigma, 93289-100ML). Complementary DNA was synthesized using a reverse transcription kit (Promega, M1705) according to the manufacturer's protocol. PCR analysis was performed using a SYBR Green PCR Mix (Bio-Rad, 1725204) by following the program: pre-denaturation at 95 ℃ for 5 min, 40 cycles of 95 ℃ for 15 s, 56℃ for 30 s, and 72℃ for 30 s. The specificity of primers was examined using melting curve. GAPDH was used as the internal reference, the relative expression of target gene was calculated using the 2^-∆∆CT^ method. The primers were synthesized by Biotechnology Co., Ltd. The primer sequences are listed in Table S1.

**Western blot analysis**

HeLa and SiHa cells were seeded in 6-well plates at a density of 1×10^6^ cells/well and treated with different concentrations of ART for 48 h. Cells were washed with PBS and lysed in RIPA lysis buffer (Beyotime, China) with protease inhibitor cocktail (Roche, Germany) and PMSF on ice, to obtain total protein. BCA protein concentration detection kit (Beyotime, China) was used to determine the protein concentration. Equal amounts of protein were separated on SDS-PAGE, and electro-transferred to polyvinylidene membranes (PVDF), then, incubated with antibodies against BCL2, BAX, Beclin-1, P62, LC3B, MMP-3, MMP-9, P-STAT3, BMI1, E-cadherin, N-cadherin, Vimentin, GAPDH, and β-actin at 4 °C. After the 3 × 10 min washes in TBST, membranes were incubated with peroxidase-conjugated secondary antibody for 1 h. Following 3 × 10 min washes in TBST, protein bands were visualized using chemiluminescent detection system.

**Table S1** **qPCR primer sequences**

| **Gene** | **Sequence** |
| --- | --- |
| *MMP-2* | Forward 5'-TCCACCAAGAAGCTGAGCGAG-3' |
|  | Reverse 5' -GTCCAGCCCATGATGGTTCT-3' |
| *MMP-3* | Forward 5'-TTCTTTGAGTTCGGTGGGGTC-3' |
|  | Reverse 5' -TGCATATTTGTTTGGGGCAGG-3' |
| *MMP-9* | Forward 5'-CATCCGGCACCTCTATGGTC-3' |
|  | Reverse 5'-CATCGTCCACCGGACTCAAA-3' |
| *MMP-13* | Forward 5'-CCCCAGGCATCACCATTCAA-3' |
|  | Reverse 5'-CATCAGGAACCCCGCATCTT-3' |
| *BMI-1* | Forward 5’-AGTGACTCTGGGAGTGACAAGG-3’ |
|  | Reverse 5’-ATTGGTGGTTACCGCTGG-3’ |
| *β-actin* | Forward 5'-CTCCTTAATGTCACGCACGAT-3' |
|  | Reverse 5' -CATGTACGTTGCTATCCAGGC-3' |

**Supplemental figures**


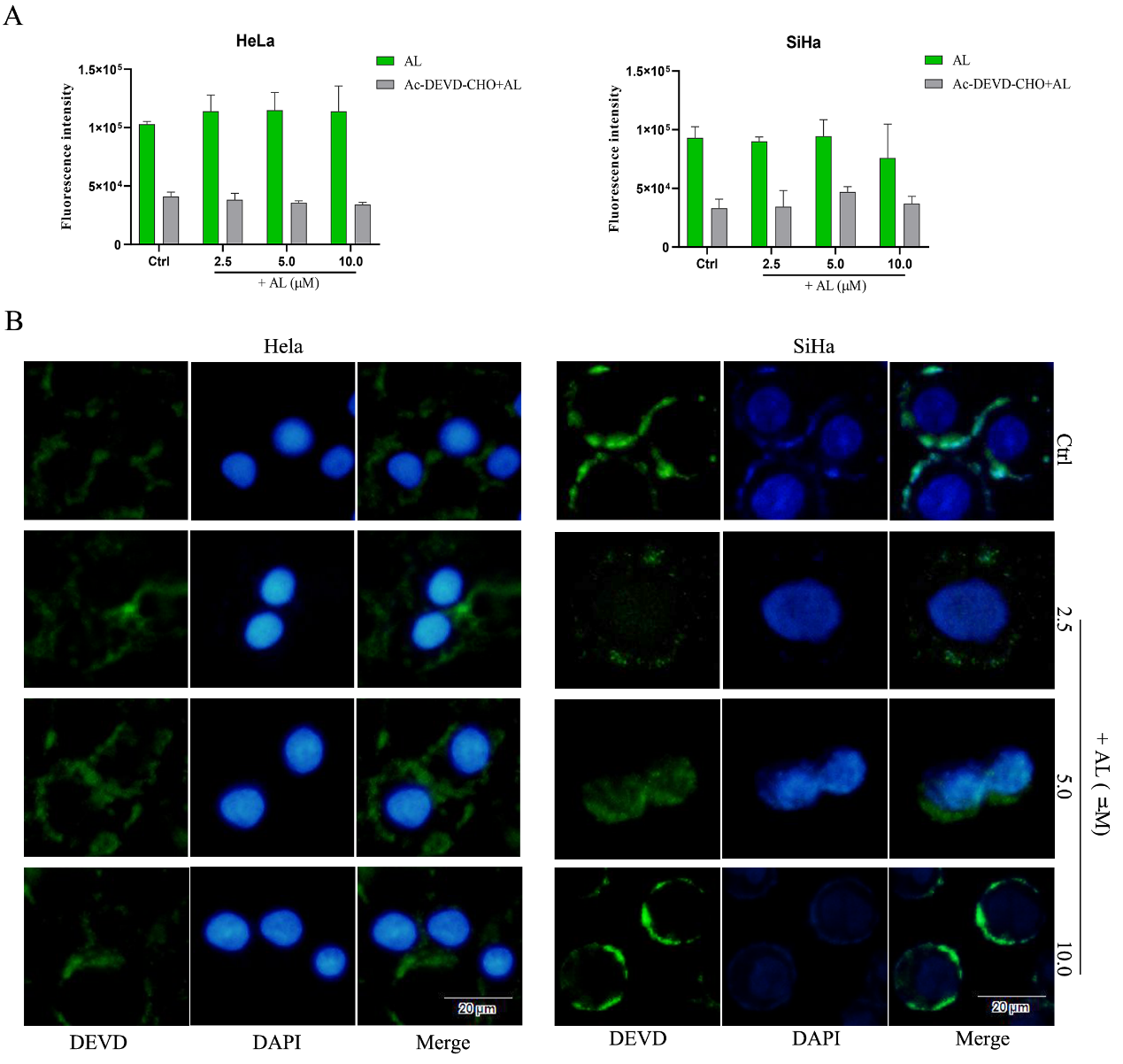


Figure S1. The inhibitory effect of AL on cervical cancer cells was caspase-3 independent. (A) AL (2.5, 5.0, 10 µM) treated HeLa and SiHa cells with or without Ac-DEVD-CHO for 24 h were stained with GreenNuc Caspase-3 Substrate, and then measured by fluorescence microplate reader or fluorescence microscope (B).


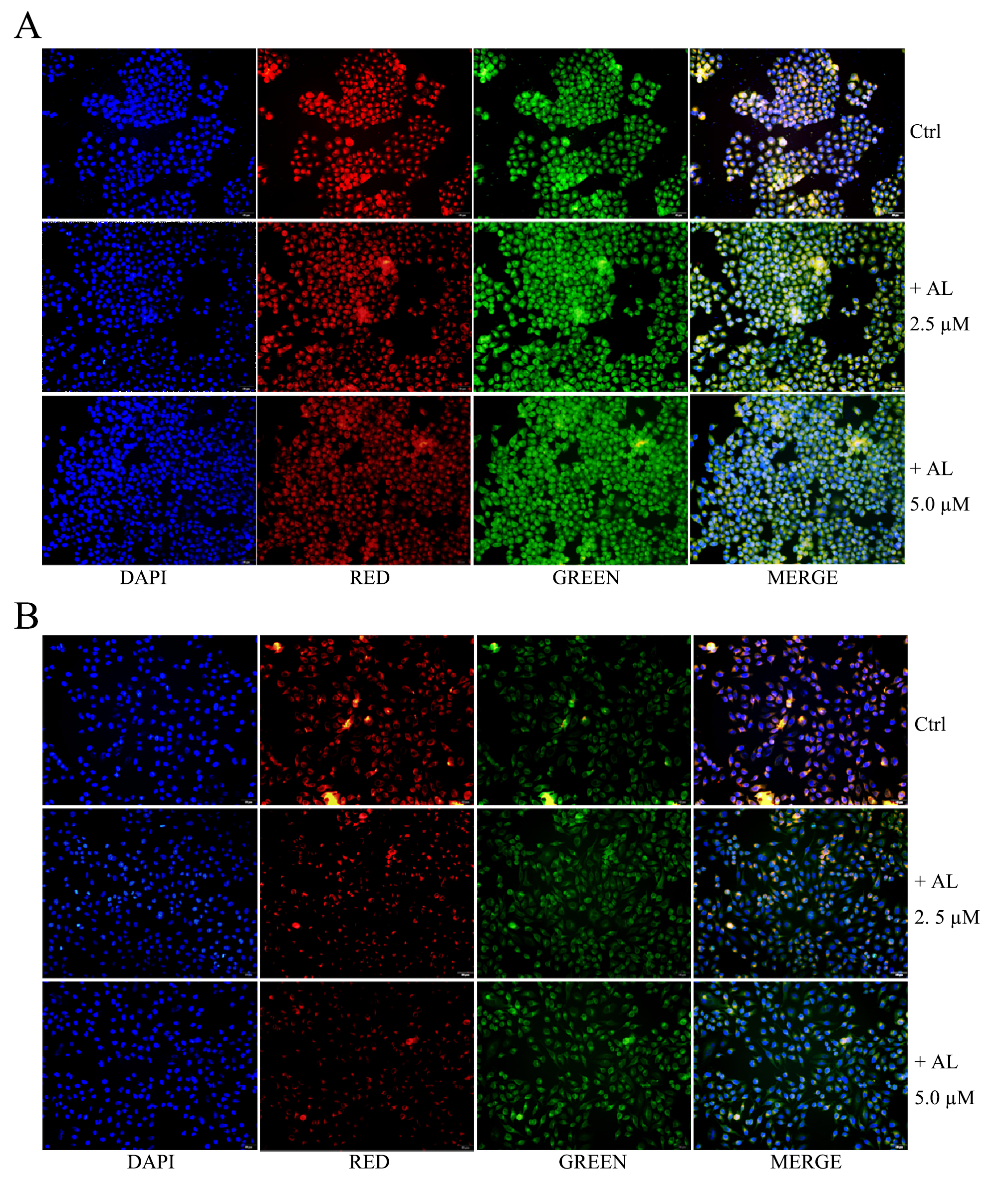


Figure S2. **AL promoted mitochondria damage in human cervical cancer cells.** HeLa (A) and SiHa (B) cells treated with AL (2.5, 5.0 µM) or vehicle for 24 h were stained with JC-1, and then measured by fluorescence microscope.


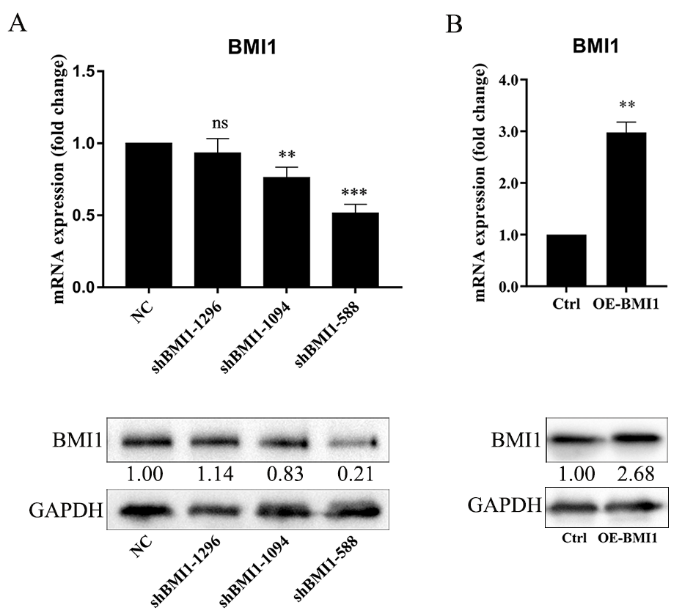


Figure S3. **BMI1 knockdown and overexpression.** (A) Knock down efficiency of three shRNA interference sequences of BMI1 were detected in HeLa cells by RT-PCR and western blot. ** *P*<0.01, *** *P*<0.001, vs NC. (B) Overexpression efficiency of BMI recombinant plasmids were detected in HeLa cells by RT-PCR and western blot. ** *P*<0.01, vs Ctrl.

**Supplementary full-length blots of main figures:**


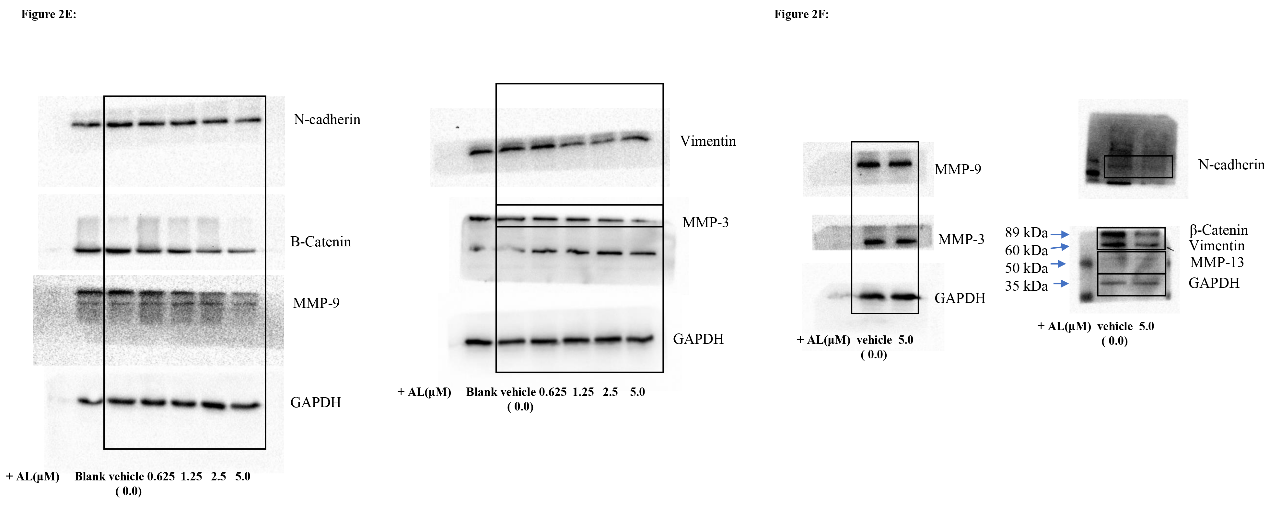


Figure 2 Equal amounts of protein were separated on SDS-PAGE, and electro-transferred to polyvinylidene membranes (PVDF), the membranes were cropped according to the molecular weight, then, incubated with different antibodies. The order of sample loading and antibodies are marked in the up figure, and the blots in the rectangular box were used in the Figure 2E and Figure 2F.


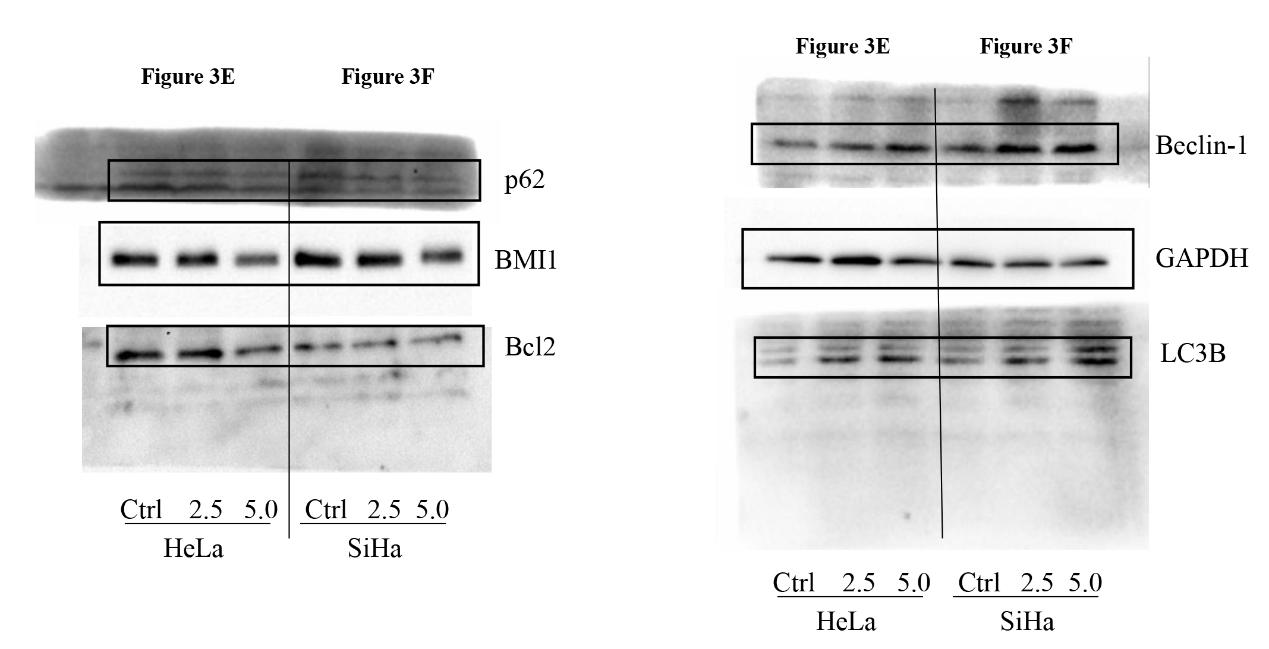


Figure 3 Equal amounts of protein were separated on SDS-PAGE, and electro-transferred to polyvinylidene membranes (PVDF), the membranes were cropped according to the molecular weight, then, incubated with different antibodies. The order of sample loading and antibodies are marked in the up figure, and the blots in the rectangular box were used in the Figure 3E and Figure 3F.


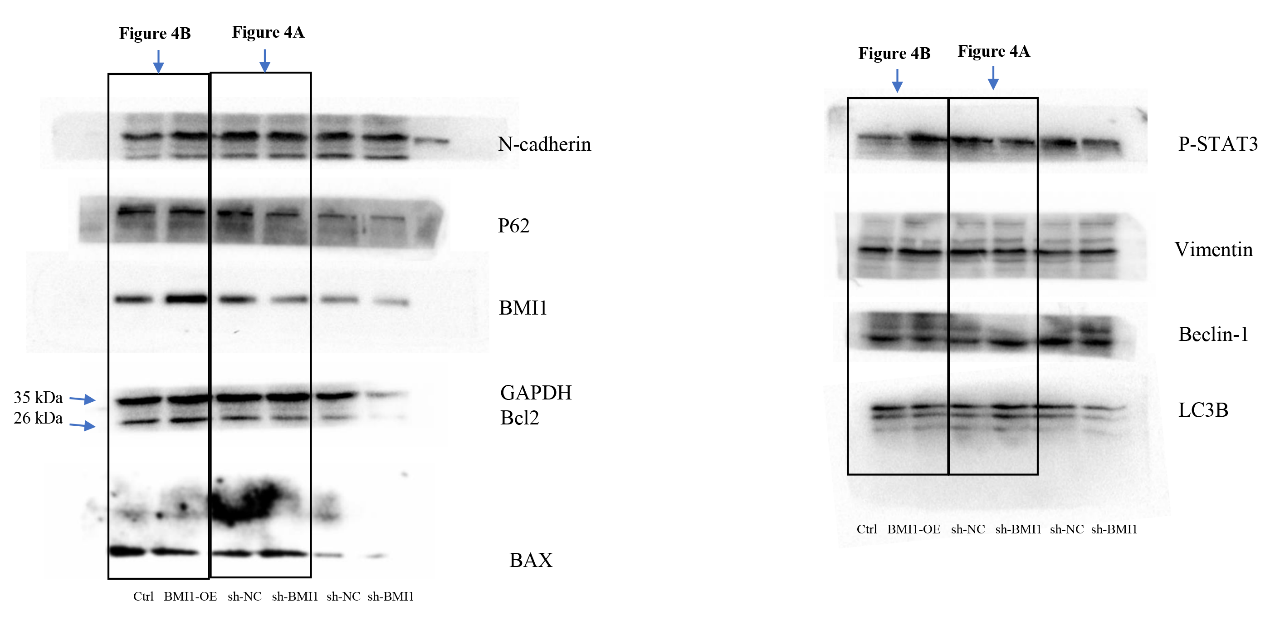


Figure 4 Equal amounts of protein were separated on SDS-PAGE, and electro-transferred to polyvinylidene membranes (PVDF), the membranes were cropped according to the molecular weight, then, incubated with different antibodies. The order of sample loading and antibodies are marked in the up figure, proteins of sh-NC group and sh-BMI1 group were extracted and detected twice, and the blots in the rectangular box were used in the Figure 4A and Figure 4B.


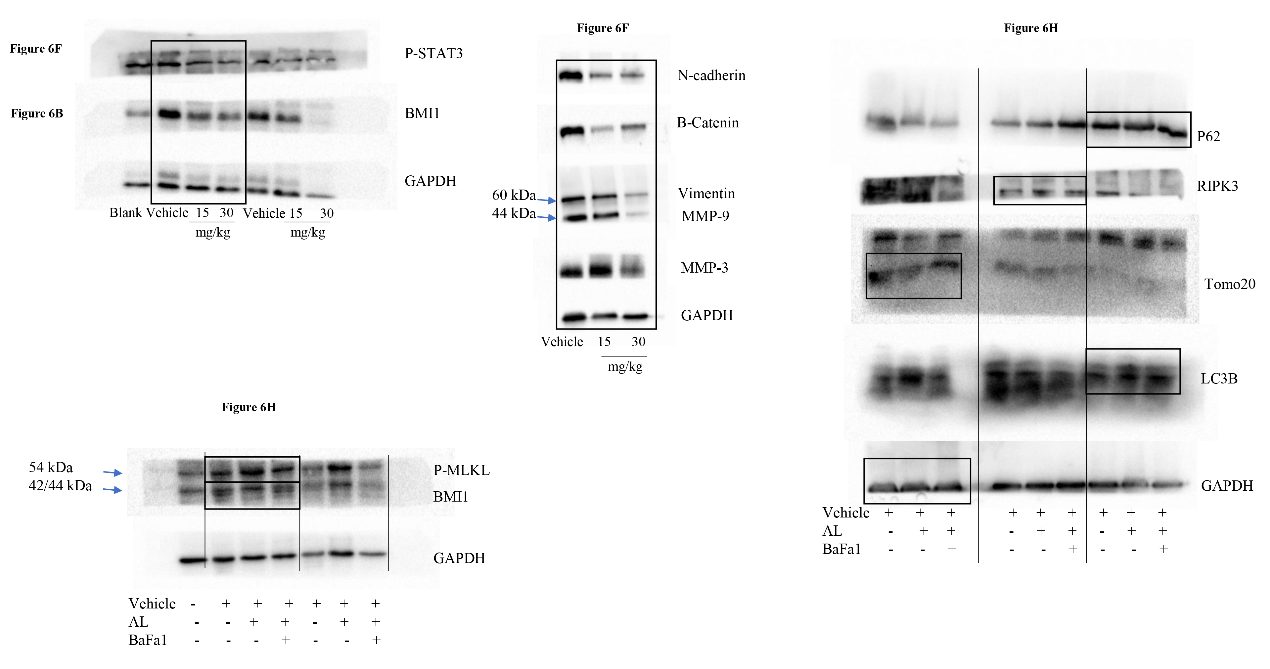


Figure 6 Equal amounts of protein were separated on SDS-PAGE, and electro-transferred to polyvinylidene membranes (PVDF), the membranes were cropped according to the molecular weight, then, incubated with different antibodies. The order of sample loading and antibodies are marked in the up figure. BMI1 and P-STAT3 expression were sampled repeatedly, and then the other proteins in Figure 6F were sampled one group. In Figure 6H, Firstly, the expression of BMI1 and P-MLKL were detected. Then, one group with consistent internal reference was selected for the subsequent detection of other proteins, and the three groups were repeated. The blots in the rectangular box were used in the Figure 6B, Figure 6F, and Figure 6H.


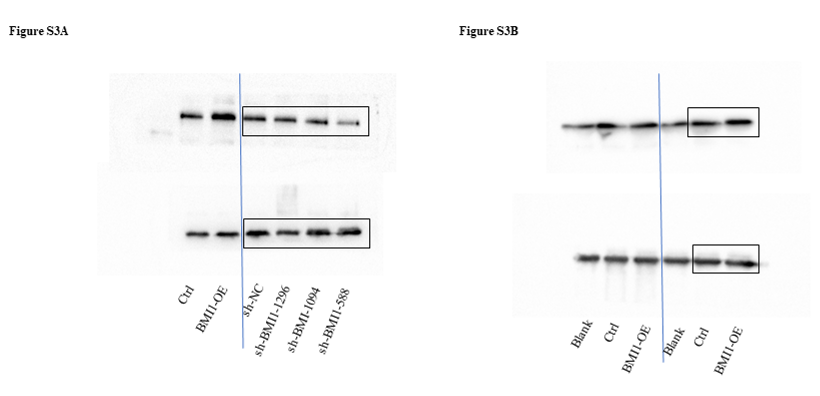


Figure S3 Equal amounts of protein were separated on SDS-PAGE, and electro-transferred to polyvinylidene membranes (PVDF), the membranes were cropped according to the molecular weight, then, incubated with different antibodies. The order of sample loading and antibodies are marked in the up figure, and the blots in the rectangular box were used in the Figure S3A and Figure S3B.
